# New Appliances and Things Medical

**Published:** 1908-04-25

**Authors:** 


### NEW APPLIANCES AND THINGS MEDICAL.

### "LOX" SAFETY PINS.

(William Oliver and Sons, 1 Winchester Avenue, Silver Street, E.C.)

To medical men and nurses safety pins are invaluable adjuncts to dressing cases properly. The ordinary domestic uses of such pins are too numerous to specify. But for doctors, as well as for other users, a safety pin, to be truly a help, should at least justify its name. The "Lox" pin does that to perfection. It is a pin that cannot possibly come open unless the user desires to unlock it. By means of a slit in the pin itself it is made to lock within the catch on a small and almost invisible point, and once locked it is absolutely secure. We have submitted the samples sent to us to various kinds of practical tests, and they have come well through the ordeal. The pins seem less likely to bend than ordinary kinds, for they are very strongly made, they can be depended upon as being absolutely safe, and are easily sterilisable. They are manufactured by the Lock Safety Pin Co., of St. Louis, U.S.A., and may be obtained from all drapers.

